# Supplementary material for: Drug repurposing for aging research using model organisms
Source: Aging Cell. 2017 Jun 16;16(5):1006–15. doi: 10.1111/acel.12626 (PMC5595691; doi:10.1111/acel.12626)
Supplement: Supplementary file 7 — Data S1 Zip‐Archive of all report cards. [file ACEL-16-1006-s007.zip › RC_B96.pdf]

B96

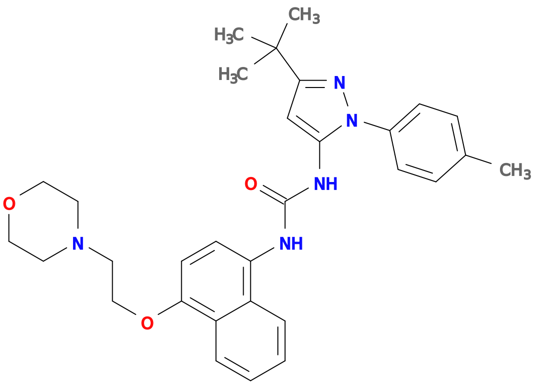

Database identifiers

ChEMBLCompound	CHEMBL103667
DrugBank	DB03044
ZINC	ZINC24044436
eMolecules	901477

Ranking

	Rank	Score
Drosophila	18/697	0.906
C. elegans	162/591	0.272

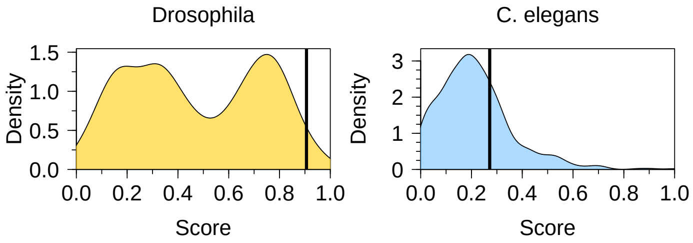

	Ageing implication	Domain conservation	Binding site conservation	Binding affinity	Bioavailability	Lipinski	Promiscuity	Purchasability	Drug approval	**Total**
Drosophila	1.0	0.971	0.992	0.963	(0.9)	-0.1	-0.004	0.1	0.075	0.906
C. elegans	1.0	0.968	0.99	0.963	0.217	-0.1	-0.004	0.1	0.075	0.272

Names

- 1-(3-(1,1-Dimethylethyl)-1-(4-methylphenyl)-1H-pyrazol-5-yl)-3-(4-(2-(morpholin-4-yl)ethoxy)naphthalen-1-yl)urea
- 1-(5-tert-butyl-2-p-tolyl-2H-pyrazol-3-yl)-3(4-(2-morpholin-4-yl-ethoxy)naphthalen-1-yl)urea
- 1-5-tert-butyl-2-(4-methylphenyl)pyrazol-3-yl-3-4-(2-morpholin-4-ylethoxy)naphthalen-1-ylurea
- 1-5-tert-butyl-2-(4-methylphenyl)pyrazol-3-yl-3-4-(2-morpholinoethoxy)-1-naphthylurea
- Doramapimod
- Urea, N-(3-(1,1-dimethylethyl)-1-(4-methylphenyl)-1H-pyrazol-5-yl)-N'-(4-(2-(4-morpholinyl)ethoxy)-1-naphthalenyl)-

Roles

ChEBI entry None has no roles

Status

Approved drug (according to ChEMBL)	No
Classification (according to DrugBank)	experimental
Number of Rule of 5 violations	2
Binding affinity to original target in log units (RF-Score prediction)	8.26
Burns *C. elegans* bioavailability prediction	-4.59

Compound Target Characteristics

Mitogen-activated protein kinase 9

Best gene implication in ageing for this target family came from gene Q8WQG9 annotated in UniProt release 2014_02. Annotation GO 8340 (determination of adult lifespan) was Inferred from Mutant Phenotype

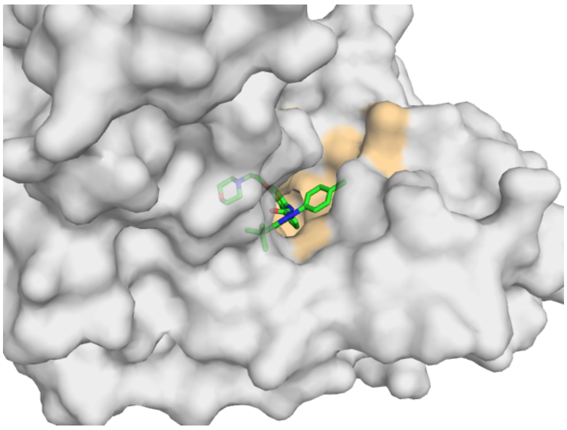

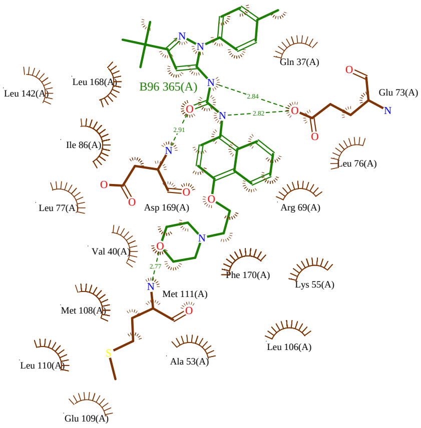

protein	amino acids contacts (binding site)																	
PDB:3npc:chainA:P45984	Q	V	A	K	R	E	L	L	I	L	M	E	L	M	L	L	D	F
tr:D7R525:D7R525_HUMAN	Q	V	A	K	R	E	L	L	I	L	M	E	L	M	L	L	D	F
tr:J3KNK1:J3KNK1_HUMAN	Q	V	A	K	R	E	L	L	I	L	M	E	L	M	L	L	D	F
sp:P45984:MK09_HUMAN	Q	V	A	K	R	E	L	L	I	L	M	E	L	M	L	L	D	F
sp:P49186:MK09_RAT	Q	V	A	K	R	E	L	L	I	L	M	E	L	M	L	L	D	F
tr:D4A5V8:D4A5V8_RAT	Q	V	A	K	R	E	L	L	I	L	M	E	L	M	L	L	D	F
sp:Q9WTU6:MK09_MOUSE	Q	V	A	K	R	E	L	L	I	L	M	E	L	M	L	L	D	F
tr:Q5NCK8:Q5NCK8_MOUSE	Q	V	A	K	R	E	L	L	I	L	M	E	L	M	L	L	D	F
tr:E1JHD6:E1JHD6_DROME	Q	V	A	K	R	E	L	M	I	L	M	E	L	M	L	L	D	F
sp:P92208:JNK_DROME	Q	V	A	K	R	E	L	M	I	L	M	E	L	M	L	L	D	F
sp:Q8WQG9:JNK1_CAEEL	Q	V	A	K	R	E	L	M	I	I	M	E	L	M	L	L	D	F
sp:P32485:HOG1_YEAST	F	V	A	K	R	E	L	L	I	F	T	E	L	Q	V	C	D	F

	whole protein		domain-based		contact-based	
protein	ident	simil	ident	simil	ident	simil
PDB:3npc:chainA:P45984	0.98	0.99	0.98	0.99	1.0	1.0
tr:D7R525:D7R525_HUMAN	0.49	0.5	0.7	0.71	1.0	1.0
tr:J3KNK1:J3KNK1_HUMAN	0.8	0.8	0.81	0.81	1.0	1.0
sp:P45984:MK09_HUMAN	1.0	1.0	1.0	1.0	1.0	1.0
sp:P49186:MK09_RAT	0.99	0.99	1.0	1.0	1.0	1.0
tr:D4A5V8:D4A5V8_RAT	0.97	0.99	0.97	0.99	1.0	1.0
sp:Q9WTU6:MK09_MOUSE	0.97	0.99	0.97	0.99	1.0	1.0
tr:Q5NCK8:Q5NCK8_MOUSE	0.99	0.99	1.0	1.0	1.0	1.0
tr:E1JHD6:E1JHD6_DROME	0.64	0.81	0.83	0.95	0.94	0.99
sp:P92208:JNK_DROME	0.64	0.81	0.83	0.95	0.94	0.99
sp:Q8WQG9:JNK1_CAEEL	0.48	0.64	0.8	0.94	0.89	0.99
sp:P32485:HOG1_YEAST	0.32	0.69	0.46	0.81	0.67	0.72

bsk (FBgn0000229) associated phenotypes

RU486 conditional, cell death defective, chemical resistant, chemical sensitive, conditional, decreased cell growth, dominant, heat sensitive, increased cell death, long lived, neuroanatomy defective, neurophysiology defective, nutrition conditional, oxidative stress response defective, planar polarity defective, progressive, short lived, somatic clone, starvation stress response defective, stress response defective, wound healing defective

(Information from FlyBase)

bsk (UniProt:P92208) annotation

Function: Responds to activation by environmental stress by phosphorylating a number of transcription factors, primarily components of AP-1 such as Jra and also the transcriptional repressor aop, and thus regulates transcriptional activity. Component of the immune response activated by bacterial infection, and is involved in wound healing and in dorsal closure, a morphogenetic movement during embryogenesis. Controls the expression of a phosphatase, puckered, at the edges of wounded epidermal tissue and in the dorsal epithelium during dorsal closure. (PubMed:10433922, PubMed:11784101, PubMed:8946915, PubMed:9224720).

Cofactor:Mg(2+)

Enzyme regulation: Activated by threonine and tyrosine phosphorylation by the dual specificity kinase, hep. Inhibited by dual specificity phosphatase, puckered. (PubMed:10433922).

Subcellular location: Cytoplasm

Tissue specificity: During gastrulation, expression is seen in cells undergoing morphogenetic movements. By stage 9 of embryonic development, expression is ubiquitous. At stages 12-14, expression occurs in epidermis and central nervous system. At stage 15, expression is restricted to ventral nerve cord, brain and some peripheral neurons. In larvae, expression is seen in all imaginal disks, with highest levels in wing and eye disks, and in the CNS. Adults express the protein in fat body and hemocytes. (PubMed:8946915, PubMed:8946916).

Developmental stage: Expressed maternally and zygotically through to adult (male and female). (PubMed:8946915).

Domain: The TXY motif contains the threonine and tyrosine residues whose phosphorylation activates the MAP kinases.

Ptm: Dually phosphorylated on Thr-181 and Tyr-183, which activates the enzyme.

(Information from UniProt)

jnk-1 (WBGene00002178) associated phenotypes

aldicarb hypersensitive, pathogen resistance increased, protein phosphorylation reduced, shortened life span, thermotolerance reduced

(Information from WormBase)

jnk-1 (UniProt:Q8WQG9) annotation

Function: Responds to activation by environmental stress by phosphorylating a number of transcription factors, and thus regulates transcriptional activity (By similarity). May coordinate locomotion by acting via type-D GABAergic motor neurons and regulate synaptic vesicle transport in conjunction with unc-16. (, PubMed:10393177, PubMed:11738026).

Cofactor:Mg(2+) Evidence=(PubMed:10393177);

Enzyme regulation: Activated by threonine and tyrosine phosphorylation by either of the dual specificity kinases, jkk-1 and sek-1. (PubMed:10393177).

Subunit: Binds to the scaffolding protein, unc-16. Unc-16 also binds other components of the JNK signaling pathway. (PubMed:11738026).

Domain: The TXY motif contains the threonine and tyrosine residues whose phosphorylation activates the MAP kinases.

Ptm: Dually phosphorylated on Thr-276 and Tyr-278, which activates the enzyme.

(Information from UniProt)

Protein-tyrosine kinase 2-beta

Best gene implication in ageing for this target family came from gene Q14289 via mapping the annotation from Ensembl ENSG00000120899 via mapping the annotation from EntrezGene 2185 via mapping the annotation from GenAgeHuman 0165 annotated in GenAge release 17.

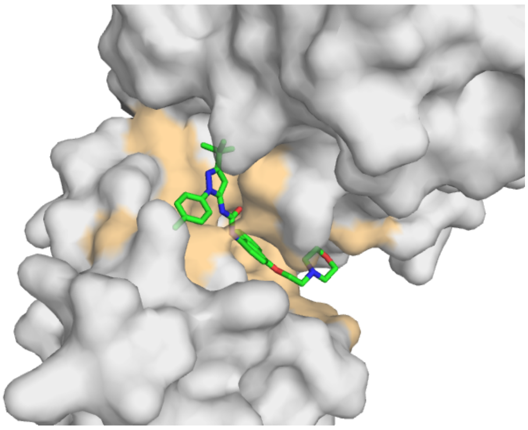

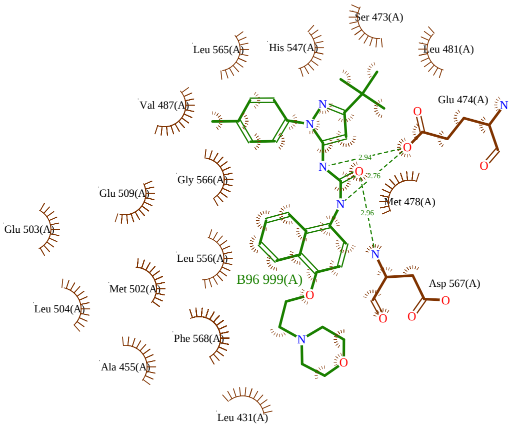

protein	amino acids contacts (binding site)																
PDB:3fzs:chainA:Q14289	L	A	S	E	M	L	V	M	E	L	E	H	L	L	G	D	F
tr:E9PBI4:E9PBI4_HUMAN	L	A	S	E	M	L	V	M	E	L	E	H	L	L	G	D	F
sp:Q14289:FAK2_HUMAN	L	A	S	E	M	L	V	M	E	L	E	H	L	L	G	D	F
sp:P70600:FAK2_RAT	L	A	S	E	M	L	V	M	E	L	E	H	L	L	G	D	F
tr:E9Q2A6:E9Q2A6_MOUSE	L	A	S	E	M	L	V	M	E	L	E	H	L	L	G	D	F
sp:Q9QVP9:FAK2_MOUSE	L	A	S	E	M	L	V	M	E	L	E	H	L	L	G	D	F
tr:Q3UDE9:Q3UDE9_MOUSE	L	A	S	E	M	L	V	M	E	L	E	H	L	L	G	D	F
tr:K7QD41:K7QD41_MOUSE	L	A	S	E	M	L	V	M	E	L	E	H	L	L	G	D	F
tr:Q2TGI0:Q2TGI0_DROME	I	A	A	E	M	F	I	M	E	L	E	H	L	L	A	D	F
tr:Q9U5Y2:Q9U5Y2_DROME	I	A	A	E	M	F	I	M	E	L	E	H	L	L	A	D	F
tr:E1JGM8:E1JGM8_DROME	I	A	A	E	M	F	I	M	E	L	E	H	L	L	A	D	F
tr:B8A418:B8A418_DROME	I	A	A	E	M	F	I	M	E	L	E	H	L	L	A	D	F
tr:Q5BIG9:Q5BIG9_DROME	I	A	A	E	M	F	I	M	E	L	E	H	L	L	A	D	F
tr:Q0E917:Q0E917_DROME	I	A	A	E	M	F	I	M	E	L	E	H	L	L	A	D	F
tr:Q9U531:Q9U531_DROME	I	A	A	E	M	F	I	M	E	L	E	H	L	L	A	D	F
tr:Q9U472:Q9U472_DROME	I	A	A	E	M	F	I	M	E	L	E	H	L	L	A	D	F
tr:B7YZL9:B7YZL9_DROME	I	A	A	E	M	F	I	M	E	L	E	H	L	L	A	D	F
tr:Q8T879:Q8T879_CAEEL	I	A	Q	E	M	F	I	L	E	L	E	H	L	L	A	D	F
tr:Q95YD4:Q95YD4_CAEEL	I	A	Q	E	M	F	I	L	E	L	E	H	L	L	A	D	F

protein	whole protein ident	whole protein simil	domain-based ident	domain-based simil	contact-based ident	contact-based simil
PDB:3fzs:chainA:Q14289	1.0	1.0	1.0	1.0	1.0	1.0
tr:E9PBI4:E9PBI4_HUMAN	0.54	0.56	1.0	1.0	1.0	1.0
sp:Q14289:FAK2_HUMAN	1.0	1.0	1.0	1.0	1.0	1.0
sp:P70600:FAK2_RAT	0.96	0.99	0.98	0.99	1.0	1.0
tr:E9Q2A6:E9Q2A6_MOUSE	0.91	0.95	0.98	0.99	1.0	1.0
sp:Q9QVP9:FAK2_MOUSE	0.95	0.99	0.98	0.99	1.0	1.0
tr:Q3UDE9:Q3UDE9_MOUSE	0.95	0.98	0.98	0.99	1.0	1.0
tr:K7QD41:K7QD41_MOUSE	0.95	0.99	0.98	0.99	1.0	1.0
tr:Q2TGI0:Q2TGI0_DROME	0.14	0.22	0.51	0.76	0.71	0.88
tr:Q9U5Y2:Q9U5Y2_DROME	0.25	0.59	0.51	0.76	0.71	0.88
tr:E1JGM8:E1JGM8_DROME	0.25	0.59	0.51	0.76	0.71	0.88
tr:B8A418:B8A418_DROME	0.25	0.59	0.51	0.76	0.71	0.88
tr:Q5BIG9:Q5BIG9_DROME	0.25	0.59	0.5	0.76	0.71	0.88
tr:Q0E917:Q0E917_DROME	0.25	0.59	0.51	0.76	0.71	0.88
tr:Q9U531:Q9U531_DROME	0.25	0.59	0.51	0.76	0.71	0.88
tr:Q9U472:Q9U472_DROME	0.25	0.6	0.51	0.76	0.71	0.88
tr:B7YZL9:B7YZL9_DROME	0.2	0.48	0.51	0.76	0.71	0.88
tr:Q8T879:Q8T879_CAEEL	0.2	0.56	0.46	0.82	0.65	0.88
tr:Q95YD4:Q95YD4_CAEEL	0.21	0.6	0.46	0.82	0.65	0.88

Fak (FBgn0020440) associated phenotypes

neuroanatomy defective, neurophysiology defective

(Information from FlyBase)

Mitogen-activated protein kinase 14

Best gene implication in ageing for this target family came from gene O62618 annotated in UniProt release 2014_02. Annotation GO 8340 (determination of adult lifespan) was Inferred from Mutant Phenotype

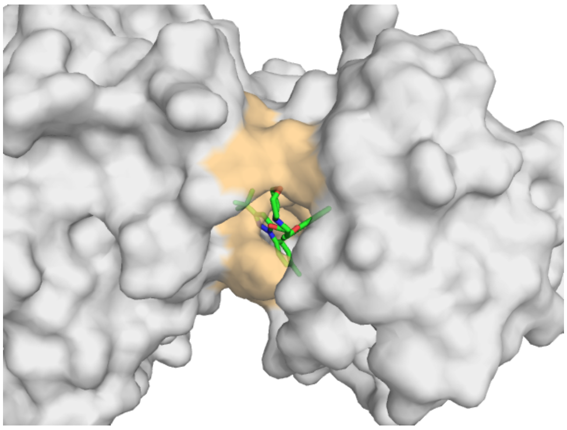

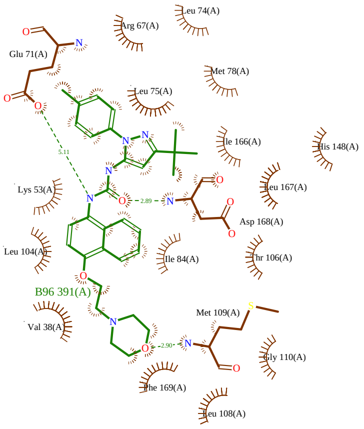

protein	amino acids contacts (binding site)
PDB:1kv2:chainA:Q16539	V K R E L L M I L T L M G H I L D F
tr:L7RSM2:L7RSM2_HUMAN	V K R E L L M I L T L M G H I L D F
sp:Q16539:MK14_HUMAN	V K R E L L M I L T L M G H I L D F
tr:G3V617:G3V617_RAT	V K R E L L M I L T L M G H I L D F
tr:Q56A33:Q56A33_RAT	V K R E L L M I L T L M G H I L D F
tr:B2KF34:B2KF34_MOUSE	V K R E L L M I L T L M G H I L D F
tr:B2KF35:B2KF35_MOUSE	V K R E L L M I L T L M G H I L D F
sp:P47811:MK14_MOUSE	V K R E L L M I L T L M G H I L D F
tr:Q5U421:Q5U421_MOUSE	V K R E L L M I L T L M G H I L D F
sp:O62618:MK14A_DROME	V K R E L L M I L T L M D H I L D F
tr:E1JIV6:E1JIV6_DROME	V K R E L L M I L T L M D H I L D F
sp:Q17446:PMK1_CAEEL	V K R E L L M I F S L M G H I L D F
sp:P32485:HOG1_YEAST	V K R E L L L I F T L Q G H I C D F

protein	whole protein ident	whole protein simil	domain-based ident	domain-based simil	contact-based ident	contact-based simil
PDB:1kv2:chainA:Q16539	1.0	1.0	1.0	1.0	1.0	1.0
tr:L7RSM2:L7RSM2_HUMAN	0.96	0.99	0.95	0.99	1.0	1.0
sp:Q16539:MK14_HUMAN	1.0	1.0	1.0	1.0	1.0	1.0
tr:G3V617:G3V617_RAT	0.99	1.0	0.99	1.0	1.0	1.0
tr:Q56A33:Q56A33_RAT	0.95	0.99	0.95	0.99	1.0	1.0
tr:B2KF34:B2KF34_MOUSE	0.56	0.56	0.62	0.62	1.0	1.0
tr:B2KF35:B2KF35_MOUSE	0.7	0.71	0.8	0.8	1.0	1.0
sp:P47811:MK14_MOUSE	0.99	1.0	0.99	1.0	1.0	1.0
tr:Q5U421:Q5U421_MOUSE	0.96	0.99	0.95	0.99	1.0	1.0
sp:O62618:MK14A_DROME	0.67	0.88	0.74	0.91	0.94	0.95
tr:E1JIV6:E1JIV6_DROME	0.67	0.88	0.74	0.91	0.94	0.95
sp:Q17446:PMK1_CAEEL	0.62	0.85	0.71	0.92	0.89	0.96
sp:P32485:HOG1_YEAST	0.41	0.68	0.54	0.85	0.78	0.83

Mpk2 (FBgn0015765) associated phenotypes

heat stress response defective, immune response defective, non-enhancer of variegation, non-suppressor of variegation, nutrition conditional, osmotic stress response defective, oxidative stress response defective, partially lethal - majority die, reduced, short lived, starvation stress response defective, stress response defective

(Information from FlyBase)

Mpk2 (UniProt:O62618) annotation

Function: Kinase involved in a signal transduction pathway. May down-regulate insect immunity gene expression after prolonged infection. (PubMed:9417090, PubMed:9584193).
Cofactor:Mg(2+)
Enzyme regulation: Activated by threonine and tyrosine phosphorylation by Mkk3 in response to environmental stress. (PubMed:9417090, PubMed:9584193).
Subcellular location: Nucleus (PubMed:9417090).
Developmental stage: Expressed both maternally and zygotically. Levels are highest at the preblastoderm stage but low levels are present throughout development. (PubMed:9584193).
Domain: The TXY motif contains the threonine and tyrosine residues whose phosphorylation activates the MAP kinases.
Ptm: Dually phosphorylated on Thr-184 and Tyr-186, which activates the enzyme. (PubMed:9417090).

(Information from UniProt)

pmk-1 (WBGene00004055) associated phenotypes

cell stress response variant, drug induced gene expression variant, gene expression level reduced, lethal, life span variant, organism oxidative stress response hypersensitive, pathogen induced gene expression variant, pathogen susceptibility increased, sterile

(Information from WormBase)

pmk-1 (UniProt:Q17446) annotation

Function: Responds to activation by environmental stress and pro- inflammatory cytokines by phosphorylating downstream targets. Functions downstream of the MAPKK sek-1 and the MAPKKK nsy-1 as the MAP kinase required for pathogen resistance. (PubMed:11703092, PubMed:12142542).

Cofactor: Mg(2+) Evidence=(PubMed:11703092);

Enzyme regulation: Activated by phosphorylation on threonine and tyrosine. Inhibited by pyridinyl-imidazole related compounds. (PubMed:11703092).

Domain: The TXY motif contains the threonine and tyrosine residues whose phosphorylation activates the MAP kinases.

Ptm: Dually phosphorylated on Thr-191 and Tyr-193, which activates the enzyme.

(Information from UniProt)
